# Measures of ability to learn, grow and make decisions among older persons: a systematic review of measurement properties

**DOI:** 10.1093/ageing/afad101

**Published:** 2023-10-30

**Authors:** Norma Mansor, Halimah Awang, Jotheeswaran Amuthavalli Thiyagarajan, Christopher Mikton, Theresa Diaz

**Affiliations:** Social Wellbeing Research Centre, Universiti Malaya, Kuala Lumpur, Malaysia; Social Wellbeing Research Centre, Universiti Malaya, Kuala Lumpur, Malaysia; Ageing and Health Unit, Department of Maternal, Newborn, Child, Adolescent Health and Ageing, World Health Organization, Geneva, Switzerland; Ageing and Health Unit, Department of Maternal, Newborn, Child, Adolescent Health and Ageing, World Health Organization, Geneva, Switzerland; Ageing and Health Unit, Department of Maternal, Newborn, Child, Adolescent Health and Ageing, World Health Organization, Geneva, Switzerland

**Keywords:** healthy ageing, functional ability, older people, psychometric properties

## Abstract

**Objective:**

this study aims to conduct a systematic review on available instruments for measuring older persons’ ability to learn, grow and make decisions and to critically review the measurement properties of the identified instruments.

**Methods:**

we searched six electronic databases, which include PubMed, Embase, PsycINFO, SciELO, ERIC and AgeLine, between January 2000 and April 2022. Reference lists of the included papers were also manually searched. The COSMIN (CONsensus-based Standards for the selection of health Measurement Instruments) guidelines were used to evaluate the measurement properties and the quality of evidence for each instrument.

**Results:**

13 instruments from 29 studies were included for evaluation of their measurement properties. Of the 13 reviewed, 6 were on the ability to learn, 3 were on the ability to grow and 4 were on the ability to make decisions. The review found no single instrument that measured all three constructs in unidimensional or multidimensional scales. Many of the instruments were found to have sufficient overall rating on content validity, structural validity, internal consistency and cross-cultural validity. The quality of evidence was rated as low due to a limited number of related validation studies.

**Conclusion:**

a few existing instruments to assess the ability to learn, grow and make decisions of older people can be identified in the literature. Further research is needed in validating them against functional, real-world outcomes.

## Key Points

- Thirteen instruments from 29 studies were included for evaluation of their measurement properties.
- No single instrument that measured all three constructs for ability to learn, grow and make decisions.
- Further research is needed in validating potential instruments against functional, real-world outcomes.

## Background

The United Nations Decade of Healthy Ageing (2021–23) calls for strengthening data and research on healthy ageing, including the measurement of older persons’ functional ability [1]. Several studies were conducted on WHO’s approach to healthy ageing [2, 3]. Greater national capacities and closer monitoring of its progress through age-disaggregated data are crucial [4]. This study focuses on the ability to learn, grow and make decisions among older people, a key component of functioning-based approach to healthy ageing [4–6]. A theoretical framework for measurement is in Appendix 1 (online supplementary materials).

The abilities to learn, grow and make decisions include efforts to continue to learn, continue personal development and be able to make choices [7]. Continuous learning equips older people with knowledge and skills; personal growth is for them to do what they value [8] while making decisions is about sense of control [7].

While there is no available literature covering all three abilities under one conceptual framework, separate studies are numerous. To our best knowledge, there has been no systematic review on the instruments to measure ability to learn, grow and make decisions in older persons [1]. This review examines the different instruments available for measuring these abilities and critically review their measurement properties. This provides reference for researchers to better prioritise their outcome measure selection for the evaluation of policies, strategies and programmes on healthy ageing.

## Method

The Preferred Reporting Items for Systematic Reviews and Meta-Analyses (PRISMA) statement guided the methodology of this systematic review. A protocol review was registered in the PROSPERO (CRD42022301165).

The WHO’s efforts to develop a monitoring and evaluation framework emphasises the importance of strengthening measurement on older persons. We conducted a systematic review of the relevant instruments in accordance with PRISMA guidelines.

### Search strategy

A comprehensive search string on related attributes for ‘learn, grow and make decisions’ was developed and ‘translated’ for searches in multiple databases from January 2022 to April 2022. Six databases were searched, which include PubMed, Embase, PsycINFO, SciELO, ERIC and AgeLine. Specific bibliographic searches of the references were also conducted to identify additional records and searches using Google Scholar’s ‘related to’ and ‘cited by’ functions for each of the articles included in the original search. The search strategy used is reported in the appendices (online Supplementary materials).

The software Microsoft Power Automate was used to automate the searches. The results were imported into Excel for screening. Duplicates and articles that do not have English titles and English full paper were removed. Study selections were carried out by two research team members to review the abstracts independently. For any discrepancy, the abstracts were reviewed by a third member to reach a decision.

### Eligibility criteria

Inclusion criteria are studies on the measurement properties of the instruments. The searches initially included studies involving older people but extended to studies with no age limit to increase the search results. Eligible studies met the following inclusion criteria: (i) full text available in English; (ii) published between 2000 and 2022 and (iii) evaluate measurement properties of any of the three abilities.

### Data collection process and data extraction

Data extracted from the reviewed studies were guided by the Cochrane Handbook for Systematic Reviews [9]. Data were captured for study purpose, population, age, instrument, measure type, number of subscales/forms and items, response options and domains measured. The CONsensus-based Standards for the selection of health Measurement Instruments (COSMIN) was used to capture data on the measurement properties and to assess the methodological quality of the studies [2, 10, 11].

### Measurement properties

Methodological quality of the selected studies was evaluated using the COSMIN taxonomy of measurement properties and definitions for health-related patient-reported outcomes. Nine psychometric properties were assessed [10, 11]. Risk of bias checklist: (i) content validity, (ii) structural validity, (iii) internal consistency, (iv) cross-cultural validity/measurement invariance, (v) measurement error, (vi) reliability (test–retest), (vii) hypothesis testing for construct validity, (xiii) criterion validity and (ix) other psychometric properties. The COSMIN guidelines were used to evaluate the measurement properties in terms of sufficient (+), insufficient (−) or indeterminate (?), or inconsistent (±). Responsiveness was outside the scope of this review while criterion validity was only evaluated for the ability to make decisions due to the absence of a ‘gold standard’ measure of ability to learn and grow. Interpretability is not considered a psychometric property under the COSMIN framework and therefore excluded. If one measurement property is rated as ‘inadequate’, the overall methodological quality is rated as ‘inadequate’, using the ‘worst count rating’ principle.

For quality of evidence, using COSMINS’s Risk of Bias [2], the pooled or summarised evidence per psychometric property was downgraded by taking the worst score counts principle on three grounds: (i) risk of bias, (ii) nonconsistency of findings across individual studies and (iii) imprecision and rated as high, moderate, low or very low.

## Results

### Systematic literature search

**Figure 1:**
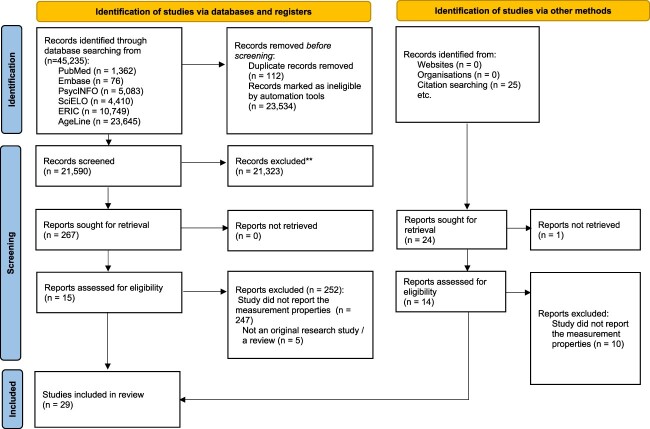
PRISMA flowchart for systematic review.

A total of 45,000 records were identified (Figure 1) with 267 shortlisted. An additional 14 articles from other searches were obtained resulting in 281 articles assessed for eligibility. Twenty-nine articles were selected for the review (Table 1) with the characteristics of instruments in Table 2.

**Table 1:** List of included studies.

Instrument	Authors	*n*	Age	Female (%)	Setting	Country (language)
Lifelong Learning Scale (LLS)	Kirby *et al*., 2010 [3]	309	>19	63.1	University students	Canada (English)
	Arslan & Ackcaalan, 2015 [12]	590	17–36	65.9	University students	Turkey (Turkish)
Lifelong Tendency Scale (LLTS)	Coskun & Demirel, 2010 [13]	642	NR	NR	Undergraduate students	Turkey (Turkish)
Effective Lifelong Learning Scale (ELLS)	Gunuc *et al*., 2014 [14]	214–528	NR	50–61	Students	Turkey (Turkish)
Self-Rating scale of Self-Directed Learning (SRSDL)	Williamson, 2007 [15]	30	20–25	NR	Nursing students	UK (English)
Work-Related Informal Learning (WRIL)	Froehlich *et al*., 2017 [16]	895	40.69^a^	60	Employees	Austria
Attitudinal Learning Inventory (ALI)	Watson *et al*., 2018 [17]	176–833	17–75	45.5–67	Students and employees	USA (English)
Personal Growth Initiative Scale-II (PGIS-II)	Robitschek *et al*., 2012 [18]	2,428	18.70–33.44^a^	64.4–70	Students and community members	USA (English)
Personal Growth and Development Scale (PGDS)	Anderson *et al*., 2019 [19]	241–468	18.16–36.9^a^	47–80	Students and employees	NR
Post-Traumatic Growth Inventory (PTGI)	Tedeschi & Calhoun, 1996 [20]	605	17–25	66.9	Undergraduate students	USA (English)
	Calhoun *et al*., 2000 [21]	54	22.5^b^	64.8	Undergraduate students	USA (English)
	Weiss, 2002 [22]	82	35–74	50	Cancer survivor and their spouses	USA (English)
	Powell *et al*., 2003 [23]	136	16–65	56.6	Individuals affected by Yugoslavian war	Yugoslavia (Bosnian)
	Sheikh & Marotta, 2005 [25]	124	64^b^	21.8	Individuals from rehabilitation programme	USA & UK (English)
	Shakespeare-Finch & Barrington, 2007 [24]	176	18–84	NR	Trauma survivors & community	Australia (English)
	Tedeschi & Calhoun, 2017 [26]	1,066	19.4–21.9^b^	49.7–75.5	Undergraduate students	USA (English), Japan (Japanese), Turkey (Turkish)
	Cann *et al*., 2010 [27] (Short PTGI)	1,351	18–85	71.9	Individuals affected by stressful events	NR
	Davey *et al*., 2015 [28]	40	20–89	NR	Middle-Eastern refugees	Australia (Arabic)
	Johnson & Boals, 2015 [29]	1,295	18–53	70.4	Undergraduate students	USA (English)
	Garcia & Wlodarczyk, 2016 (Short PTGI) [30]	1,817	18–84	53.9	Individuals affected by stressful events	Chile (Chilean)
	Li *et al*., 2021 [31]	124	24–79	66.9	Caregivers of people with dementia	China (Chinese)
Decision Making among Older Adults (DMC-OA)	Finucane & Gullion, 2010 [33]	608	25–97	62.5	General community	USA (English)
Assessment of Capacity for Everyday Decision-Making (ACED)	Lai *et al*., 2008 [34]	52	62–81^b^	NR	Outpatient and caregivers	USA (English)
	Lui *et al*., 2013 [35]	275	>60	82	Older residents	Hong Kong (NR)
Making Everyday Decisions for Safe and Independent Living (MED-SAIL)	Mills *et al*., 2014 [36]	49	76^b^	57.1	Outpatient geriatrics clinic	USA (English)
	Mills *et al*., 2020 [40]	24	68.2	NR	Nursing home residents	USA (English)
Adult Decision-Making Competence (ADMC)	De Bruin *et al*., 2007 [37]	360	18–88	73.8	Social service and community group	USA (English)
	Bavolar, 2013 [38]	508	18–26	62.6	Students	Slovakia (Slovak)
	Peng *et al*., 2019 [39]	560	19–41	39.1	Education institutions, and the army	China (Chinese)

^a^Median

^b^Mean

NR, figure not reported.

**Table 2:** Characteristics of instruments.

Ability measured	Instrument	Type of instrument	Dimensions and items	Time required to administer	Available in public domain	Interviewer
Learn	Lifelong Tendency Scale (LLTS) [14]	Self-reported	Motivation, perseverance, lack of regulating learning, lack of curiosity; 74 items	NR	Instrument is copyrighted and freely available	None
	Lifelong Learning Scale (LLS) [12, 13]	Self-reported	Goal setting, application of knowledge and skills; self-direction and evaluation; locating information; adaptable learning strategies; 14 items	NR	Instrument is copyrighted and freely available	None
	Effective Lifelong Learning Scale (ELLS) [15]	Self-reported	Attitude towards learning, self-evaluation for learning, motivation towards learning, management and planning of learning process, skills and competences; 48 items	NR	Instrument is copyrighted and freely available	None
	Self-Rating scale of Self-Directed Learning (SRSDL) [16]	Self-reported	Awareness, learning strategies, learning activities, evaluation, interpersonal skills; 60 items	NR	Instrument is copyrighted and freely available	None
	Work-Related Informal Learning (WRIL) [17]	Self-reported	Feedback seeking, information seeking, social support; 12 items	NR	Instrument is copyrighted and freely available	None
	Attitudinal Learning Inventory (ALI) [18]	Self-reported	Cognitive learning, affective learning, behavioural learning, social learning; 15 items	NR	Instrument is copyrighted and freely available	None
Grow	Personal Growth and Development Scale (PGDS) [20]	Self-reported	Autonomy; environmental mastery; positive relations; self-acceptance purpose in life; 15 items	30 min	Instrument is copyrighted and freely available	None
	Personal Growth Initiative Scale-II (PGIS-II) [19]	Self-reported	Planfulness; using resources; readiness for change; intentional behaviour; 16 items	NR	Instrument is copyrighted and freely available	None
	Post-Traumatic Growth Inventory (PTGI) [21–32]	Self-reported	New possibilities; relating to others; personal strength, spiritual change; appreciation of life; existential and spiritual change (PTGI-X); 21–25 items	NR	Instrument is copyrighted and freely available	None
Make Decisions	Decision Making among Older Adults (DMC-OA) [34]	Face-to-face interview	Comprehension; consistency; dimension weighting; cognitive reflection 45 items	45–90 min	Instrument is copyrighted and freely available	Trained interviewer
	Assessment of Capacity for Everyday Decision-Making (ACED) [35, 36]	Face-to-face interview	Ability to understand; ability to appreciate; ability to reason; ability to express a choice; 6 items	15–20 min	Instrument is copyrighted and freely available	NR
	Making Everyday Decisions for Safe and Independent Living (MED-SAIL) [37, 38]	Face-to-face interview	Understanding; appreciation; expressing a choice; reasoning; generating consequences; 5 items	15 min	Instrument is copyrighted and partially available	Trained interviewer
	Adult Decision-Making Competence (ADMC) [39–41]	Face-to-face interview	Resistance to framing; recognising social norms; under/overconfidence; applying decision rules; consistency in risk perception; resistance to sunk costs; path independence; 87 items	NR	Instrument is copyrighted and freely available	NR

NR, not reported.

### Characteristics of included studies and instruments

Of the 29 articles reviewed, seven were related to the ability to learn, 14 ability to grow and eight ability to make decisions. Studies were conducted in Europe (Austria, UK, Yugoslavia, Slovakia, Turkey), North America (Canada, USA), Asia Pacific (Australia, Japan, China and Hongkong) and South America (Chile). Studies on the ability to learn mostly involved young respondents in universities, while studies on ability to grow and make decisions covered slightly older respondents (age 17–97) [3, 12–39]. Six studies were conducted in clinical settings. The studies for ability to learn and grow were self-reported assessment while the instruments for ability to make decisions used face-to-face interviews [24, 31, 34–36, 40].

The instruments on the ability to learn consisted of the Lifelong Learning Scale (LLS) [3, 12], Lifelong Tendency Scale (LLTS) [13], Effective Lifelong Learning Scale (ELLS) [14], Self-Rating scale of Self-Directed Learning (SRSDL) [15] and Work-Related Informal Learning (WRIL) [16]. The LLS study involved 309 university students [3] aged ≥19 years with 63% female. The instrument was translated into Turkish [12], which involved 590 university students aged 17–36, with 66% female. A study on LLTS [13] covered 642 undergraduate students in Turkey while Gunuc *et al*. [14] focused on ELLS involving multiple groups of 214–528 students with 50–60% female. SRSDL [15] covered a small sample of 30 nursing students aged 20–25, while WRIL [16] focused on 895 employees with an average age of 41 years and 60% female. Attitudinal Learning Inventory (ALI) [17] instrument was administered on multiple groups of 176–833 students and employees aged 17–75, 46–67% female.

Instruments on the ability to grow consisted of Personal Growth Initiative Scale-II (PGIS-II) [18], Personal Growth and Development Scale (PGDS) [19] and Post-Traumatic Growth Inventory (PTGI) [21–31]. The original form of PGIS [18, 32], not included in this review, was further improved and developed through PGIS-II. The PGIS-II [18] involved 2,428 students and community members with mean age 18.70–33.44 and 64.4–70% female while PGDS [19] involved multiple groups of 241–468 students and employees with mean age 18.16–36.9, 47–80% female.

The PTGI was developed [20] involving 605 undergraduate students aged 17–25 with 67% female. The instrument was validated in Calhoun *et al.* [21] using 54 undergraduate students with an average age of 22.5 years, 65% female and another study [22] that involved 41 cancer survivors and their respective spouses aged 35–74. Subsequently, the instrument was translated into Bosnian language [23] involving 136 individuals aged 16–65 affected by the Yugoslavian War comprising 57% female. The instrument was also administered to 124 individuals with a history of heart diseases attending rehabilitation programme [24], where their average age was 64 years with 22% female. Another study [25] involved 176 individuals who were trauma survivors and community members aged 18–84. The PTGI was further improved and validated in Tedeschi *et al.* [26] by adding the spiritual component to their original instrument. The study involved 1,066 undergraduate students from the USA, Turkey and Japan with mean age 19.4–21.9 comprising 50–76% female. Cann *et al.* [27] conducted a study using 1,351 individuals aged 18–85 affected by stressful events with 72% female. There were other studies validated using the original PTGI involving 40 Middle-Eastern refugees [28] aged 20–89 and another involving 1,295 undergraduate students [29] aged 18–53 with 71% female. Another big study involving 1,817 Chileans affected by stressful events [30] aged 18–84 with 54% female. A recent study using PTGI [31] involved 124 family caregivers of people with dementia aged 24–79 with 67% females.

The instruments to measure the ability to make decisions consisted of Decision Making among Older Adults (DMC-OA), Assessment of Capacity for Everyday Decision-Making (ACED), Making Everyday Decisions for Safe and Independent Living (MED-SAIL) and Adult Decision-Making Competence (ADMC). DMC-OA was developed by Finucane and Gullion [33] involving 608 community members aged 25–97 comprising 63% female. A study [34] developed the ACED involving 52 outpatients and caregivers with mean age 62–81. The instrument was validated in a study by Lui *et al.* [35] based on 275 older residents aged ≥60 years in Hong Kong with 82% female. Another study [36] developed MED-SAIL involving 49 outpatients in a geriatrics clinic with an average age of 76 years with 57% female. The authors improved the instrument using 24 nursing home residents with an average age 68.2 years [40]. ADMC was developed by De Bruin *et al.* involving 360 social service and community groups aged 18–88 with 74% female and later translated and validated by other studies in Slovakia and China [15, 39], in which the study in Slovakia involved 508 students aged 18–26 with 63% female while the China study involved 560 individuals aged 19–41 from education institutions and the army, with 39% female.

### Measurement properties and quality of the evidence

Table 3 presents the overall rating of the psychometric properties of the three abilities and the quality of evidence based on COSMIN’s Risk of Bias Checklist [2]. Measurement properties that were not reported or evaluated in the selected studies were regarded as ‘no information provided’. None of the included studies reported measurement error.

#### Ability to learn

None of the nine studies on the ability to learn addressed content validity in terms of its relevance, comprehensiveness and comprehensibility. All six scales were applied to the relevant sample involving students and/or employees [3, 13–17]. Content validity of LLTS, ELLS and SRSSDL was tested using experts’ opinions [13–15]. Overall, there was sufficient but low quality of evidence for content validity. This is due to the single validation study conducted on each instrument with the exception for LLS, where two validation studies were conducted.

Four instruments were assessed on structural validity using Exploratory Factor Analysis (EFA) and Confirmatory Factor Analysis (CFA). LLTS was validated using only EFA [13]. Overall rating was sufficient for these instruments, quality of evidence for LLS [3, 12] was moderate as both studies confirmed single factor structure while the other instruments were low due to validation by a single study [13, 14, 16, 17].

Five instruments exhibited reasonably high internal consistency in full scale or subscales with a Cronbach’s alpha ≥ 0.7 [13, 14, 16, 17]. The LLS showed an inconsistent Cronbach’s alpha in two studies [3, 12]. The overall rating was sufficient but with low quality of evidence, due to inconsistent Cronbach’s alpha [3, 12], and validation was done by a single study [13, 14, 16, 17].

Two instruments were assessed on cultural validity. The LLS has been translated into Turkish [12], which was originally in English. Even though the WRIL was validated in one study [16], the factor analysis was conducted using different heterogenous subsamples. The overall rating was sufficient with moderate quality of evidence.

For construct validity, only the SRSSDL was assessed via known-group validity where discrepancy was found between two distinct groups [15]. The overall rating was sufficient, but with low quality of evidence due to a single study. The instruments of LLS [3] and ALI [17] were assessed on convergent validity. Both instruments exhibited some correlations with some other variables. The overall rating was sufficient with low quality of evidence for both studies.

#### Ability to grow

Three instruments were developed based on comprehensive and relevant existing theories and literature [19, 20, 24]. The overall rating was sufficient, but quality of evidence was only high for PTGI.

For structural validity, a four-factor structure was found in PGIS-II [18] and single factor structure in PGDS [19]. However, the two instruments were validated by a single study. The PTGI developed by [20] was validated with five-factor structure in seven studies [20–22, 27, 28, 31, 32] and four-factor, three-factor and single factor found in a single study [15, 25, 29]. The EFA and CFA were performed for each instrument. The overall rating for the PGIS-II and PGDS were sufficient but with low quality of evidence due to a single study. The overall rating for PTGI was sufficient with moderate quality of evidence based on multiple studies.

Cronbach’s alpha for assessing internal consistency for overall scales and/or subscales of PGIS-II and PGDS were ≥0.7 [18, 19]. However, the quality of evidence was low due to validation by a single study. For the PTGI, the Cronbach’s alpha for overall scales and/or subscales of five-factor structure and four-factor structure were ≥0.7 [20–22] while the three-factor structure was lower [25]. Overall rating was sufficient with high quality of evidence.

Cross-cultural validity for the PGIS-II [24] and PGDS [19] was assessed across respondents with diverse backgrounds using factor analysis. For the PTGI, factor analysis was performed across different languages, cultures, regions, countries and traumatic events [15, 22, 25, 27–32]. The overall rating was sufficient with moderate quality of evidence for PGIS-II and PGDS and high for PTGI.

Reliability test–retest was performed on all three instruments [18–20]. Pearson correlations for reliability test–retest of the PGIS-II and PGDS were 0.62–0.82 and 0.71 for PTGI. The overall rating for the PGIS-II and PGDS was sufficient with low quality of evidence while the overall rating for PTGI was sufficient with moderate quality of evidence.

Convergent validity was assessed for the three instruments [18–20, 25, 30–32] and found to have some correlations with other measures. The overall rating was sufficient while quality of evidence was moderate for PGIS-II and PGDS and high for PTGI.

**Table 3:** List of instruments included in the review.

Measurement property	Summary or pooled results	Overall rating	Quality of evidence
Lifelong Learning Scale			
Content validity	No information available		
Structural validity	Single-factor structure [3, 12]	Sufficient (+)	Moderate: two good studies
	Total sample size: 899		
Predictive validity	No information available		
Internal consistency	Internal consistency [3, 12]	Indeterminate (?)	Low: two good studies inconsistency was found across the two
	Cronbach’s alpha values of two studies: 0.67–0.77		
	Total sample size: 899		
Cross-cultural validity/measurement invariance	Single-factor structure [3, 12]	Sufficient (+)	Moderate: two good studies
	Qualitative summary across countries and languages		
	Total sample size: 899		
Reliability (test–retest)	No information available		
Measurement invariance	No information available		
Criterion validity	No information available		
Convergent validity	Qualitative pooling for convergent validity (pooled correlation coefficients) [3]	Sufficient (+)	Low: single good study
	Lifelong learning and deep learning: 0.43		
	Lifelong learning and surface learning: −0.36		
	Total sample size: 309		
Other properties	No information available		
Lifelong Learning Tendency Scale			
Content validity	Content validity [13]	Sufficient (+)	Low: single good study
	Relevance	Sufficient (+)	Low: single good study
	Comprehensiveness	Sufficient (+)	Low: single good study
	Comprehensibility	Sufficient (+)	Low: single good study
Structural validity	4-factor structure [13]	Sufficient (+)	Low: single good study
	Total sample size: 642		
Predictive validity	No information available		
Internal consistency	Internal consistency [13]	Sufficient (+)	Low: single good study
	Cronbach’s alpha value: 0.89		
	Total sample size: 642		
Cross-cultural validity/measurement invariance	No information available		
Reliability (test–retest)	No information available		
Measurement invariance	No information available		
Criterion validity	No information available		
Convergent validity	Lifelong learning tendency and curiosity index: 0.67	Sufficient (+)	Low: single good study
	Total sample size: 642		
Other properties	No information available		
Effective Lifelong Learning Scale			
Content validity	Content validity [14]	Sufficient (+)	Low: single good study
	Relevance	Sufficient (+)	Low: single good study
	Comprehensiveness	Sufficient (+)	Low: single good study
	Comprehensibility	Sufficient (+)	Low: single good study
Structural validity	1-factor structure	Sufficient (+)	Low: single good study
	Total sample size: 742		
Predictive validity	No information available		
Internal consistency	Internal consistency [14]	Sufficient (+)	Low: single good study
	Cronbach’s alpha values: 0.96		
	Total sample size: 742		
Cross-cultural validity/measurement invariance	No information available		
Reliability (test–retest)	No information available		
Measurement invariance	No information available		
Criterion validity	No information available		
Convergent validity	No information available		
Other properties	No information available		
Work-Related Informal Learning			
Content validity	No information available		
Structural validity	4-factor structure [16]	Sufficient (+)	Low: single very good study
	Total sample size: 895		
Predictive validity	No information available		
Internal consistency	Internal consistency [16]	Sufficient (+)	Low: single very good study
	Cronbach’s alpha values of subscales: 0.658–0.863		
	Total sample size: 895		
Cross-cultural validity/measurement invariance/measurement invariance	4-factor structure [16]	Sufficient (+)	Moderate: single very good study
	Qualitative summary across work organisations and nationalities		
	Total sample size: 895		
Reliability (test–retest)	No information available		
Measurement error	No information available		
Criterion validity	No information available		
Convergent validity	No information available		
Other properties	No information available		
Self-Directed Learning			
Content validity	Content validity [15]	Sufficient (+)	Low
	Relevance	Sufficient (+)	Low
	Comprehensiveness	Sufficient (+)	Low
	Comprehensibility [15]	Sufficient (+)	Low
Structural validity	5-factor structure	Insufficient (−)	Low: no factor analysis was performed
	Total sample size: 30		
Predictive validity	No information available		
Internal consistency	Internal consistency [15]	Sufficient (+)	Low: single good study
	Cronbach’s alpha values of subscales: 0.71–0.79		
	Total sample size: 30		
Cross-cultural validity/measurement invariance	No information available		
Reliability (test–retest)	No information available		
Measurement invariance	No information available		
Criterion validity	No information available		
Convergent validity	Known-group validity [15]	Sufficient (+)	Low: single good study
	Final year students’ scores are higher than first year students		
	Total sample size: 30		
Other properties	No information available		
Attitudinal Learning			
Content validity	No information available		
Structural validity	4-factor structure [17]	Sufficient (+)	Low: single very good study
	Total sample size: 1,009		
Predictive validity	No information available		
Internal consistency	Internal consistency [17]	Sufficient (+)	Low: single very good study
	Cronbach’s alpha values of subscales: 0.79–0.95		
	Total sample size: 1,009		
Cross-cultural validity/measurement invariance	No information available		
Reliability (test–retest)	No information available		
Measurement invariance	No information available		
Criterion validity	No information available		
Convergent validity	Qualitative pooling for convergent validity (pooled correlation coefficients) [17]	Sufficient (+)	Low: single very good study
	Affective learning and final exam: 0.17		
	Affective learning and certificate delivered: 0.22		
	Social learning and final exam: 0.22		
	Total sample size: 833		
Other properties	No information available		
Personal Growth Initiatives			
Content validity	Content validity [18]	Sufficient (+)	Moderate
	Relevance	Sufficient (+)	Moderate
	Comprehensiveness	Sufficient (+)	Moderate
	Comprehensibility	Sufficient (+)	Moderate
Structural validity	4-factor structure [18]	Sufficient (+)	Moderate: single very good study
	Total sample size: 2,428		
Predictive validity	No information available		
Internal consistency	Internal consistency [18]	Sufficient (+)	Moderate: single very good study
	Cronbach’s alpha values of subscales: 0.79–0.88		
	Total sample size: 2,428		
Cross-cultural validity/measurement invariance	4-factor structure [18]	Sufficient (+)	Moderate: single very good study
	Qualitative summary across students with diverse backgrounds and community members		
	Total sample size: 2,428		
Reliability (test–retest)	Subgroup explanation of study with time interval ranges from 1 to 6 weeks [18]	Sufficient (+)	Moderate: single very good study
	Pearson’s correlation coefficient of study: 0.62–0.82		
	Total sample size: 2,428		
Measurement invariance	No information available		
Criterion validity	No information available		
Convergent validity	Qualitative pooling for convergent validity (pooled correlation coefficients) [18]	Sufficient (+)	Moderate: single good study
	PGIS-II and Original PGIS: 0.20–0.57		
Other properties (concurrent validity)	No information available		
Personal Growth and Development Scale			
Content validity	Content validity [19]	Sufficient (+)	Low
	Relevance	Sufficient (+)	Low
	Comprehensiveness	Sufficient (+)	Low
	Comprehensibility	Sufficient (+)	Low
Structural validity	Single factor structure [19]	Sufficient (+)	Low: single good study
	Total sample size: 1,086		
Predictive validity	No information available		
Internal consistency	Internal consistency [19]	Sufficient (+)	Low: single good study
	Cronbach’s alpha: 0.90		
	Total sample size: 1,086		
Cross-cultural validity/measurement invariance	Single-factor structure [19]	Sufficient (+)	Moderate: single good study
	Qualitative summary across students and employees		
	Total sample size: 1,086		
Reliability (test–retest)	Subgroup explanation of study with time interval ranges from time 1 to 2 [19]	Sufficient (+)	Low: single good study
	Pearson’s correlation coefficient of study: 0.65		
	Total sample size: 1,086		
Measurement error	No information available		
Criterion validity	No information available		
Convergent validity	Qualitative pooling for convergent validity (pooled correlation coefficients) [19]	Sufficient (+)	Moderate: single good study
	PGDS and SPWB: 0.53		
	PGDS and BNS-G: 0.62		
	PGDS and MBI: −0.47		
	PGDS and UWES: 0.56		
	PGDS and Pos Aff: 0.66		
	PGDS and Neg Aff: −0.34		
	PGDS and PHQ: −0.14		
Other properties	No information available		
Post-traumatic Growth Inventory			
Content validity	Content validity		
	Relevance	Sufficient (+)	High
	Comprehensiveness	Sufficient (+)	High
	Comprehensibility	Sufficient (+)	High
Structural validity	5-factor structure [20–22, 27, 30, 31] Total sample size: 5,284	Sufficient (+)	Moderate: multiple very good studies with inconsistent results
	4-factor structure Total sample size: 40		
	3-factor structure [23] Total sample size: 136		
	Single factor [24] Total sample size: 124		
Internal consistency	5-factor structure [20–22, 25, 27, 29–31] Cronbach’s alpha: 0.89–0.97 Total sample size: 5,284	Sufficient (+)	High: multiple very good studies
	4-factor structure [28] Cronbach’s alpha: 0.94 Total sample size: 40		
	3-factor structure [23] Cronbach’s alpha of subscales: 0.547–0.61 Total sample size: 136		
Cross-cultural validity	Cross-cultural validity [20, 22–24, 27–31] Performed factor analysis across subgroups Translated into different languages Applied across cultures, regions and countries Across different traumatic events	Sufficient (+)	High: multiple very good studies
Reliability (test–retest)	Subgroup explanation of studies with time interval of around 2 months [20] Pearson’s correlation coefficients of studies: 0.71 Sample size: 604	Sufficient (+)	Moderate: covered in only 1 study
Measurement error	No information available		
Criterion validity	No information available		
Hypothesis testing for construct validity	PTGI correlated with other measures [20, 23, 29–31] PTGI and Neo Personality Inventory: 0.16–0.29 Corrected PTG and Changes in self/positive life attitude: 0.610 Corrected PTG and Philosophy of life: 0.571 Corrected PTG and Relating to others: 0.547 PTGI and Gratitude: 0.23–0.44 PTGI and Pos relations: 0.14–0.49 PTGI and satisfaction with life: 0.18–0.51 PTGI and religious: 0.17–0.39 PTGI and meaning life: 0.22–0.40 PTGI and C-PTGI: 0.38–0.55 PTGI and DASS: −0.43 PTGI-SF and SSE: 0.51 PTGI-SF and IES-R: 0.42 PTGI and Family functioning: 0.47 PTGI and Positive coping strategies: 0.26 PTGI and Negative coping strategies: −0.21	Sufficient (+)	High: multiple very good studies
Responsiveness	No information available		
Others	No information available		
Decision Making among Older Adults			
Content validity	Content validity [32]	Sufficient (+)	Low: single good study
	Relevance		
	Comprehensiveness		
	Comprehensibility		
	Total sample size: 608		
Structural validity	Three-factor structure [32] 27.5% variance	Insufficient (−)	Low: single good study
	Total sample size: 608		
Internal consistency	Three-factor structure [32]	Sufficient (+)	Low: single good study
	Cronbach’s alpha subscale value: 0.62–0.80		
	Total sample size: 608		
Cross-cultural validity	Three-factor structure	Sufficient (+)	Low: single good study
	Quantitative study between different age group		
	Total sample size: 608		
Reliability	No information provided		
Measurement error	No information provided		
Criterion validity	No information provided		
Hypothesis testing for construct validity	Quantitative pooling for convergent validity (pooled correlation coefficients)	Sufficient (+)	Low: single good study
	Total sample size: 608		
Other properties	Discriminant validity Concurrent validity Moderate, positive associations with related construct	Sufficient (+)	Low: single good study

Making Everyday Decisions for Safe and Independent Living			
Content validity	Content validity	Sufficient (+)	High: multiple good studies with consistent result
	Relevance [36, 40]		
	Comprehensiveness [36, 40]		
	Comprehensibility		
	Total sample size: 73		
Structural validity	Four-factor structure	Insufficient (−)	Low: multiple studies with insufficient results
	Qualitative summary: no CFA is performed		
	Total sample size: 73		
Internal consistency	4-factor structure [36, 40]	Sufficient (+)	Moderate
	Cronbach’s alpha value: 0.77–0.78 with mean of 0.85		
	Total sample size: 73		
Cross-cultural validity	4-factor structure [36, 40]	Insufficient	Low
	Qualitative summary: hypothesis supported		
	Total sample size: 73		
Reliability	No information		
Measurement error	No information		
Criterion validity	AUC > 0.70 [36]	Sufficient (+)	Moderate
	Total sample size: 49		
Hypothesis testing for construct validity	Quantitative pooling for convergent validity [36, 40] MED-SAIL and ILS: 0.573 MED-SAIL and IADL: 0.440 MED-SAIL and MoCA: 0.72 Total sample size: 73	Sufficient (+)	High
Other properties	Concurrent validity [36] Responsiveness [36] Divergent validity [36] Total sample size: 49	Sufficient (+)	Moderate: multiple studies with inconsistent results
Assessment of Capacity for Everyday Decision-Making			
Content validity	Content validity	Sufficient (+)	High
	Relevance [37–39]		
	Comprehensiveness		
	Comprehensibility		
Structural validity	Four-factor structure [37–39]	Sufficient (+)	Low
	Total sample size: 327		
Internal consistency	Four-factor structure [37–39]	Sufficient (+)	High
	Cronbach’s alpha values		
	Total sample size: 327		
Cross-cultural validity	Four-factor structure [37–39]	Sufficient (+)	High
Reliability	Subgroup explanation [37–39]	Sufficient (+)	High
	Total sample size: 327		
Measurement error	No information		
Criterion validity	No information		
Hypothesis testing for construct validity	Quantitative pooling for convergent validity [37–39] Spearman −0.18 to 0.22 Correlation with ACED and MMSE <0.48 < *r* < 0.60	Sufficient (+)	High
	Total sample size: 327		
Other properties	No information		
Adult Decision-Making Competence			
Content validity	Content validity	Sufficient (+)	High
	Relevance		
	Comprehensiveness		
	Comprehensibility		
Structural validity	Two-factor structure	Inconsistent (±)	Low: multiple good studies but inconsistent result
	Total sample size: 1,428		
Internal consistency	Two-factor structure	Sufficient (+)	High
	Total sample size: 1,428		
Cross-cultural validity	Two-factor structure	Sufficient (+)	High
	Qualitative summary of studies		
	Total sample size: 1,428		
Reliability	Subgroup explanation: interval of 1–9 days Interval of 1 month	Sufficient (+)	Moderate: multiple good studies
	Total sample size: 920		
Measurement error	No information		
Criterion validity	No information		
Hypothesis testing for construct validity	No information		
Other properties	No information		

SPWB, Composite Scales of Psychological Well-Being; BNS-G, Basic Needs Satisfaction in General Composite; MBI, Maslach Burnout Inventory (Student version); UWES, Utrecht Work Engagement Survey (Student version); Pos Aff, Positive Dimension of the Positive and Negative Affect Schedule; Neg Aff, Negative Dimension of the Positive and Negative Affect Schedule; PHQ, Physical Health Questionnaire; ILS, Independent Living Scale; IADL, Instrumental Activities of Daily Living; MoCA, Monaco Cognitive Assessment; MMSE, Mini-Mental State Examination.

#### Ability to make decisions

ADMC included professionals or experts to consider its relevancy and comprehensibility related to content validity [37–39]. MED-SAIL instrument asked patients regarding relevancy of its items [36, 37]. Overall rating was sufficient with moderate to high quality of evidence. Low quality of evidence was observed for DMC-OA as the instrument was validated by a single study.

A four-factor structure was used in DMC-OA, MED-SAIL and ACED while the ADMC used a two-factor structure. Low quality of evidence for structural validity in all four instruments was due to inconsistent results or small sample size. ADMC and DMC-OA sufficiently performed EFA or CFA, while MED-SAIL and ACED did not perform any factor analysis; ACED had better overall rating due to a larger sample size compared to MED-SAIL.

The qualitative summary for internal consistency had sufficient methodological quality. Cronbach’s alpha for DMC-OA subscales was 0.62–0.82, while for MED-SAIL, both studies [36, 40] registered Cronbach’s alpha averaging 0.85. Multiple studies using ACED and ADMC measures had Cronbach’s alpha 0.54–0.78 and 0.54–0.79, respectively. All four instruments were found to have sufficient rating for internal consistency with moderate to high quality of evidence except for DMC-OA as it was based on a single study.

For cross-cultural validity, all instruments except for DMC-OA were validated in multiple studies across various groups. While the DMC-OA was administered to a large sample size with a wide age range, it was only conducted in one setting. ACED and MED-SAIL were conducted in clinical settings involving participants with no cognitive to full cognitive performance. ADMC had a high overall rating as the measure was administered across countries, yielding a high quality of evidence regarding measurement invariance.

The reliability test–retest was performed on ACED and ADMC. Intraclass correlation coefficient (ICC) was calculated for ACED yielding a high-quality evidence with the Spearman correlation coefficient 0.65–0.92 while test–retest was performed on ADMD with different intervals, resulting in ICC 0.28–0.77. The other two measures did not report information on reliability validity. Overall rating ACED and ADMC was sufficient with moderate to high quality of evidence.

For construct validity, the quantitative summary was found to be sufficient for all three instruments, except for ADMC. Concurrent validity was assessed for MED-SAIL, yielding significant correlations with Independent Living Scales (ILS), Instrumental Activities of Daily Living (IADL) and Montreal Cognitive Assessment (MoCA); the coefficients were 0.440–0.72. Correlations between ACED and Mini-Mental State Examination (MMSE) were 0.48–0.60. Concurrent validity was confirmed as DMC-OA had moderate association with related constructs. No information was provided for construct validity on ADMC. Overall rating was sufficient with high quality of evidence except for MED-SAIL, which only has a single study.

The criterion validity was assessed on MED-SAIL using area under the receiver operating characteristic curve (AUC) [36] with AUC >0.7. MED-SAIL was assessed for responsiveness and divergent validity and found significant.

## Discussion

### Summary of key findings

This study conducted a systematic review of the ability to learn, grow and make decisions that constitutes one of the functional abilities to measure and monitor healthy ageing. There are numerous studies on the ability to learn, grow and make decisions that represent the general population; however, there are too few studies available to develop and validate the abilities with specific focus on older people. No studies were done to develop and validate the three abilities in a single, uniformed instrument. The studies included in this review were not limited to older people only and include wide range of sample as to maximise the number of instruments to be evaluated. Instruments with specific focus on the ability to learn and grow were conducted mostly in university settings, where the participants invariably possess a certain degree of cognitive functioning, while the ability to make decisions seems to be particularly focused on cognitive capacity and assessment in clinical settings.

#### Ability to learn

Our review showed that the ability to learn emphasised learning that took place not in the formal setting, although the sample involved were largely university students and employees. This indicates that learning was initiated by self-motivation and the quest for further knowledge and skills. Many of the measures on the ability to learn explicitly assess behaviours towards learning in universities or workplace involving younger participants. There are several original research on these measures that have not been validated by other independent research. In this review, four instruments were found to sufficiently address the required psychometric properties with low to moderate quality of evidence. However, only LLS [3, 12] was validated by more than one study. This raises concern with respect to increased risk of bias. Moreover, the measures assessed used the lifelong learning concepts, where learning cannot be confined to formal institutions, but rather can take place in a wide variety of settings—workplace, voluntary associations, social and recreational contexts—encompassing formal, non-formal and informal education. Lifelong learning is more appealing in the context of older adults.

#### Ability to grow

PTGI [20–31] is an instrument that is most studied and validated compared to other two scales PGIS and PGDS. While the PTGI has been administered to a wide range of age groups and in different settings involving large sample sizes, the instrument requires life-changing events such as illnesses, loss of loved ones or war that trigger some degree of acceptance and growth. Such events may be of less concern to the larger population. PTGI and PGDS were demonstrated to have potential to be used as in a large survey; however, these instruments suffer from increased risk of bias as they were not validated by other researchers.

#### Ability to make decisions

For the ability to make decisions, the instruments developed were majorly based on older adults and conducted in clinical settings where participants with no to partial capacity were also assessed. Hence, these instruments are suitable to be administered in a large survey. Overall, ACED and ADMC were found to have better measurement properties compared to the other two instruments; however, as ADMC has 80 items that may be too long, the former instrument is more suitable to be used in a large-scale study due to its high quality, portability and short completion time. MED-SAIL suffers the most in terms of risk of bias as it is the only single study used to assess the ability to make decisions, and while the measured properties showed a largely low quality of evidence.

## Conclusion

The ability to learn, grow and make decisions is one of the domains of function ability in the approach to healthy ageing adopted by the WHO Decade of Healthy Ageing [5]. The review shows that there is no single instrument that addresses all three abilities. The questions arise as to whether there is a need to redesign all these instruments and combine them to become one uniform instrument. However, in many instances, these abilities seemed to follow a somewhat logical sequence where one develops and grows from the experience of learning, which in turn provides the reasoning and justifications to make informed decisions. While these instruments addressed some selected psychometric properties, more studies need to be conducted to validate the instrument, particularly in a more general setting involving older adults.

## Supplementary Material

aa-23-0344-File002_afad101
