# Supplementary material for: Measures of ability to learn, grow and make decisions among older persons: a systematic review of measurement properties
Source: Age Ageing. 2023 Oct 30;52(Suppl 4):iv118–32. doi: 10.1093/ageing/afad101 (PMC10615040; doi:10.1093/ageing/afad101)
Supplement: aa-23-0344-File002_afad101 [file aa-23-0344-file002_afad101.docx]

World Health Organization: *Measurement of Healthy Ageing.*

**Measures of ability to learn, grow and make decisions among older persons: A systematic review of measurement properties.**

SUPPLEMENTARY DATA

- **Appendix 1:** Search terms.
- **Appendix 2:** List of included and excluded studies.
  - **Appendix 2a:** List of included studies.
  - **Appendix 2b:** List of excluded studies.
- **Appendix 3:** Potential measures / Questionnaire.

**Appendix 1: Search terms***

(MH " education") OR (MH " learning") OR (MH "practicing") OR (MH "Grow") OR (MH " Growth") OR (MH "making decisions") OR (MH "Skills") OR (MH "knowledge") OR (MH "literacy") OR (MH "development") OR (MH "applying") OR (MH " experience") OR (MH " Coping") OR (MH " adapting") OR ( life-long adj learning. tw)

Search terms validation studies:

(MH "Psychometrics") OR (MH "Measurement Issues and Assessments") OR (MH "Validity") OR (MH "Predictive Validity") OR (MH "Reliability and Validity") OR (MH "Internal Validity") OR (MH "Face Validity") OR (MH "External Validity") OR (MH "Discriminant Validity") OR (MH "Criterion-Related Validity") OR (MH "Consensual Validity") OR (MH "Concurrent Validity") OR (MH "Qualitative Validity") OR (MH "Construct Validity") OR (MH "Content Validity") OR (MH "Questionnaire Validation") OR (MH "Validation Studies") OR (MH "Test-Retest Reliability") OR (MH "Sensitivity and Specificity") OR (MH "Reproducibility of Results") OR (MH "Reliability") OR (MH "Intrarater Reliability") OR (MH "Interrater Reliability") OR (MH "Measurement Error") OR (MH "Bias (Research)") OR (MH "Selection Bias") OR (MH "Sampling Bias") OR (MH "Precision") OR (MH "Sample Size Determination") OR (MH "Repeated Measures") OR (Psychometric* or reliability or validity* or reproducibility or bias)))

Search terms for older people:

((exp Aged/ OR (senior$1 or elderly or older).tw) OR (MH " Frail elderly") OR (exp aged/ or middle aged/) OR ((old* or age*) adj3 (people* or person* or adult* or women* or men* or citizen* or residen*)) OR (pension* or retire* or elderly or senior* or geriatric*)

Exclusion

NOT (‘delphi-technique’[ti] OR cross-sectional[ti] OR "addresses"[Publication Type] OR "biography"[Publication Type] OR "case reports"[Publication Type] OR "comment"[Publication Type] OR "directory"[Publication Type] OR "editorial"[Publication Type] OR "festschrift"[Publication Type] OR "interview"[Publication Type] OR "lectures"[Publication Type] OR "legal cases"[Publication Type] OR "legislation"[Publication Type] OR "letter"[Publication Type] OR "news"[Publication Type] OR "newspaper article"[Publication Type] OR "patient education handout"[Publication Type] OR "popular works"[Publication Type] OR "congresses"[Publication Type] OR "consensus development conference"[Publication Type] OR "consensus development conference, nih"[Publication Type] OR "practice guideline"[Publication Type]) NOT ("animals"[MeSH Terms] NOT "humans"[MeSH Terms])

*Search term may be adjusted for different databases

**Appendix 2: List of included and excluded studies.**

**Appendix 2a: List of included studies.**

| **No** | **Author** | **Title** |
| --- | --- | --- |
| 1 | Anderson et al., 2020. | Measuring Personal Growth and Development in Context: Evidence of Validity in Educational and Work Settings |
| 2 | Arslan & Ackcaalan, 2015. | The Adaptation and Validation of the Turkish Version of the Lifelong Learning Scale (LLS). |
| 3 | Bavolar, 2013. | Validation of the Adult Decision-Making Competence in Slovak students |
| 4 | de Bruin, 2007. | Individual differences in adult decision-making competence |
| 5 | Calhoun et al., 2000. | A Corretional test of the relationship between posttraumatic growth, religion, and cognitive processing |
| 6 | Cann et al., 2010 | A short form of the posttraumatic growth inventory. Anxiety, Stress and Coping |
| 7 | Coşkuna & Demirel 2010. | Lifelong learning tendency scale: The study of validity and reliability |
| 8 | Davey et al., 2015 | Development of the Arabic versions of the Impact of Events Scale-Revised and the Posttraumatic Growth Inventory to assess trauma and growth in Middle Eastern refugees in Australia. |
| 9 | Finucane & Gullion 2010. | Developing a Tool for Measuring the Decision-Making Competence of Older Adults |
| 10 | Froehlich, 2017. | Development and validation of a scale measuring approaches to work-related informal learning |
| 11 | García & Wlodarczyk, 2016. | Psychometric Properties of the Posttraumatic Growth Inventory–Short Form Among Chilean Adults |
| 12 | Gunuc et al., 2015. | Developing an effective lifelong learning scale (ELLS): Study of validity and reliability |
| 13 | Kirby et al. | Development of a scale to measure lifelong learning |
| 14 | Johnson & Boals | Refining our ability to measure posttraumatic growth. Psychological Trauma: Theory, Research, Practice, and Policy |
| 15 | Lai et al., 2008 | Everyday decision-making ability in older persons with cognitive impairment |
| 16 | Li et. al, 2020 | Coping strategies mediate the association between family functioning and posttraumatic growth in family caregivers of people with dementia |
| 17 | Lui et al. | Structured assessment of mental capacity to make financial decisions in Chinese older persons with mild cognitive impairment and mild Alzheimer disease |
| 18 | Mills et al., 2020 | Validation of the MEDSAIL Tool to Screen for Capacity for Safe and Independent Living Among Nursing Home Residents. |
| 19 | Mills et al., 2014 | Making and executing decisions for safe and independent living (MED-SAIL): Development and validation of a brief screening tool |
| 20 | Peng et al., 2013 | Measuring decision-making competence in Chinese adults |
| 21 | Powell et al., 2003 | Posttraumatic growth after war: A study with former refugees and displaced people in Sarajevo |
| 22 | Robitschek, 2012. | Development and Psychometric Evaluation of the Personal Growth Initiative Scale–II |
| 23 | Shakespeare-Finch & Barrington, 2007. | Behavioural Changes Add Validity to the Construct of Posttraumatic Growth |
| 24 | Sheikh & Marotta, 2005. | A cross-validation study of the Posttraumatic Growth Inventory |
| 25 | Tedeschi et al., 2007 | The Posttraumatic Growth Inventory: A Revision Integrating Existential and Spiritual Change |
| 26 | Tedeschi & Calhoun. | The posttraumatic growth inventory: Measuring the positive legacy of trauma |
| 27 | Watson, 2018 | The development and validation of the Attitudinal Learning Inventory (ALI): a measure of attitudinal learning and instruction |
| 28 | Weiss, 2002 | Posttraumatic growth in women with breast cancer and their husbands: An intersubjective validation study |
| 29 | Williamson, S. N. | Development of a self-rating scale of self-directed learning |

**Appendix 2b:** List of excluded studies

| **No** | **Authors** | **Title** |
| --- | --- | --- |
| 1 | Abrams et al., 2019 | The Interview for Decisional Abilities (IDA): a tool to assess the decisional capacity of abused and neglected older adults |
| 2 | Al-Omari, 2013 | The Relationship between Decision Making Styles and Leadership Styles among Public Schools Principals |
| 3 | Alexander et al., 2014 | The Impact of Project-Based Learning on Pre-Service Teachers' Technology Attitudes and Skills |
| 4 | Ali et al., 2021 | Aging and Engaging: A Pilot Randomized Controlled Trial of an Online Conversational Skills Coach for Older Adults |
| 5 | Atchley, 2006 | Continuity, Spiritual Growth, and Coping in Later Adulthood |
| 6 | Backman et al., 2020 | Shared Decision-Making for Older Adults with Cardiovascular Disease |
| 7 | Bailey et al., 2013 | Age-Related Differences in Social Economic Decision Making: The Ultimatum Game |
| 8 | Bailly et al., 2012 | Coping with negative life events in old age: The role of tenacious goal pursuit and flexible goal adjustment |
| 9 | Barrera et al., 2015 | Cognitive-behavioral therapy for late-life anxiety: Similarities and differences between Veteran and community participants |
| 10 | Barry et al., 2018 | Assessment of Mental Capacity and Decision-Making |
| 11 | Begley et al., 2019 | How are people with dementia involved in care-planning and decision-making? An Irish social work perspective |
| 12 | Bellebaum et al., 2012 | Positivity effect in healthy aging in observational but not active feedback-learning |
| 13 | Ben-Ari et al., 2012 | Personal growth and meaning in life among first-time mothers and grandmothers. |
| 14 | Bhatt et al., 2020 | The nature of decision-making in people living with dementia: a systematic review |
| 15 | Biella et al., 2020 | Decision-Making Profile in Older Adults: The Influence of Cognitive Impairment, Premorbid In intelligence and depressive symptoms |
| 16 | Bir et al., 2021 | Does Education Change Elderly’s Attitude Towards Old Age and Decision-Making? |
| 17 | Black et al., 2013 | Decision Making for Participation in Dementia Research |
| 18 | Bolenz et al., 2019 | Metacontrol of Decision-Making Strategies in Human Aging |
| 19 | Boulton-Lewis & Buys, 2014 | Older Academics: Motivation to Keep Working |
| 20 | Boulton-Lewis et al., 2016 | Hong Kong and Australian seniors: Views of aging and learning. |
| 21 | Boulton-Lewis et al., 2017 | Ageing, loss, and learning: Hong Kong and Australian seniors. |
| 22 | Boyd et al., 2019 | Decision Making for Older Adults With Multiple Chronic Conditions: Executive Summary for the American Geriatrics Society Guiding Principles on the Care of Older Adults With Multimorbidity |
| 23 | Brennan et al., 2012 | Coping trajectories in later life: A 20-year predictive study |
| 24 | Brittain et al., 2012 | Perceived Barriers, Coping Strategies, and Self-Regulatory Efficacy to Cope: An Examination of Sufficiently and Insufficiently Physically Active Middle-Aged and Older Women |
| 25 | Bruce et al., 2015 | Clinical effectiveness of integrating depression care management into medicare home health: the Depression CAREPATH Randomized trial |
| 26 | Bryanton & Weeks, 2014 | Informing the Development of Educational Programs to Support Older Adults in Retiring from Driving |
| 27 | Bugos, 2014 | Adult Learner Perceptions: Perspectives from Beginning Musicians (Ages 60-86 Years) |
| 28 | Cadorin et al., 2020 | Evaluating Self-Directed Learning Abilities as a Prerequisite of Health Literacy among Older among older people: Findings from a validation and a cross-sectional study |
| 29 | Cameron et al., 2017 | Development of a decision-making tool for reporting drivers with mild dementia and mild cognitive impairment to transportation administrators |
| 30 | Carbone, 2021 | Supporting route learning in older adults: the role of imagery strategy |
| 31 | Casemiro et al., 2018 | Effects of health education in the elderly with mild cognitive impairment |
| 32 | Castro & Dolores, 2021 | Barriers and supportive factors for older adults learning about and using information and communication technologies for healthy aging in Costa Rica |
| 33 | Chaffin, 2006 | Cognitive learning applied to older adult learners and technology |
| 34 | Chang & Sung, 2011 | Motivation to Learn among Older Adults in Taiwan |
| 35 | Charles et al., 2021 | Decision‐making capacity assessment education |
| 36 | Chen et al., 2012 | The K-shape Learning Project for Senior Citizens |
| 37 | Chen et al., 2014 | Age Differences in Adaptive Decision Making: The Role of Numeracy |
| 38 | Chi et al., 2017 | Multimorbidity and Decision-Making Preferences among Older Adults |
| 39 | Chiu et al., 2016 | The attitudes, impact, and learning needs of older adults using apps on touchscreen mobile devices: Results from a pilot study. |
| 40 | Choi et al., 2012 | Examining the aging process through the stress-coping framework: application to driving cessation in later life |
| 41 | Coady, 2013 | Adult health learning and transformation: A case study of a Canadian community-based program. |
| 42 | Cooper et al., 2017 | Framing Matters: Effects of Framing on Older Adults’ Exploratory Decision-Making |
| 43 | Cordella et al., 2012 | Intergenerational and Intercultural Encounters: Connecting Students and Older People Through Language Learning |
| 44 | Cordova et al., 2001 | Posttraumatic growth following breast cancer: A controlled comparison study |
| 45 | Craciun, 2012 | Successful Aging-Utopia or the Result of Lifelong Learning? Meaning and Representations of Ageing in Romanian Elderly |
| 46 | Cruz & Herzog, 2018 | Setting the Faculty on Fire: Fostering Vitality in Late Career Faculty |
| 47 | Curran et al., 2019 | Adult Learners' Perceptions of Self-Directed Learning and Digital Technology Usage in Continuing Professional Education: An Update for the Digital Age |
| 48 | Danhauer et al., 2012 | Predictors of posttraumatic growth in women with breast cancer. |
| 49 | Danhauer et al., 2013 | A longitudinal investigation of posttraumatic growth in adult patients undergoing treatment for acute leukaemia. |
| 50 | Davis et al., 2017 | Everyday Decision Making in Individuals with Early-Stage Alzheimer's Disease: An Integrative Review of the Literature |
| 51 | de Bruin et al., 2014 | Getting Older Isn't All That Bad: Better Decisions and Coping When Facing "Sunk Cost" |
| 52 | Del Missier et al., 2020 | Decision-Making Competence in Older Adults: A Rosy View from a Longitudinal Investigation |
| 53 | Dinaj-Koci et al., 2015 | Adolescent Sexual Health Education: Parents Benefit Too! |
| 54 | Donnelly et al., 2019 | 153 Barriers and Enablers of Assisted Decision-making for Older People in Acute Care Hospitals: A Multi-Stakeholder Inquiry |
| 55 | Drago-Severson et al., 2013 | Learning and Leading for Growth: Preparing Leaders to Support Adult Development in Our Schools |
| 56 | Duff et al., 2013 | Learning in Alzheimer's disease is facilitated by social interaction |
| 57 | Easton et al., 2013 | Posttraumatic growth among men with histories of child sexual abuse. |
| 58 | Elvish et al., 2014 | ‘Getting to Know Me’: the development and evaluation of a training programme for enhancing skills in the care of people with dementia in general hospital settings |
| 59 | Endedijk, 2014 | Students' Development in Self-Regulated Learning in Postgraduate Professional Education: A Longitudinal Study |
| 60 | Engel et al., 2015 | Delivery of self training and education for stressful situations (DESTRESS-PC): a randomized trial of nurse assisted online self-management for PTSD in primary care |
| 61 | Eppinger et al., 2012 | Reduced Sensitivity to Immediate Reward during Decision-Making in Older than Younger Adults |
| 62 | Escolar-Chua & de Guzman, 2014 | Effects of third age learning programs on the life satisfaction, self-esteem, and depression level among a select group of community dwelling Filipino elderly. |
| 63 | Fatima et al., 2020 | Age, executive functioning, and decision-making styles in adults: a moderated mediation model |
| 64 | Feldman, 2012 | The Impact of Intergenerational Volunteering and Learning |
| 65 | Fetherstonhaugh et al., 2013 | Being central to decision making means I am still here!: The essence of decision making for people with dementia |
| 66 | Garand et al., 2014 | Effects of problem-solving therapy on mental health outcomes in family caregivers of persons with a new diagnosis of mild cognitive impairment or early dementia: a randomized controlled trial |
| 67 | Gitlin et al., 2014 | Factors mediating the effects of a depression intervention on functional disability in older African Americans |
| 68 | Gladman, 2019 | Personal growth and development in old age—a clinician's perspective |
| 69 | Gooding et al., 2012 | Psychological resilience in young and older adults |
| 70 | Gould et al., 2017 | Hoping for the Best or Planning for the Future: Decision Making and Future Care Needs |
| 71 | Gumport et al., 2018 | Patient learning of treatment contents in cognitive therapy |
| 72 | Gyselinck et al., 2013 | Considering spatial ability in virtual route learning in early aging. |
| 73 | Hachem & Vuopala, 2016 | Older adults, in Lebanon, committed to learning: Contextualizing the challenges and the benefits of their learning experience. |
| 74 | Han et al., 2015 | Mild Cognitive Impairment Is Associated with Poorer Decision-Making in Community-Based Older Persons |
| 75 | Han et al., 2016 | Discrepancies between Cognition and Decision Making in Older Adults |
| 76 | Hansen et al., 2020 | Enhancing older adult access to lifelong learning institutes through technology-based instruction: A brief report |
| 77 | Harper, 2014 | Development of a Health Literacy Assessment for Young Adult College Students: A Pilot Study |
| 78 | Hauer et al., 2012 | Developmental intervention, learning climate and use of knowledge in elderly care. |
| 79 | Hayslip, Bert | Lifelong Learning: A Dyadic Ecological Perspective |
| 80 | Hennekam, 2015 | Career success of older workers: The influence of social skills and continuous learning ability. |
| 81 | Heo et al., 2017 | Leisure activities, optimism, and personal growth among the young-old, old-old, and oldest-old. |
| 82 | Hermann et al., 2017 | Measuring perceptions of the learning environment and approaches to learning: Validation of the Learn Questionnaire. |
| 83 | Hess et al., 2019 | Use of Descriptive and Experiential Information in Decision Making by Young and Older Adults |
| 84 | Heuer et al., 2020 | Service-Learning with older adults in care communities: Measures of attitude shifts in undergraduate students |
| 85 | Holden et al., 2018 | Understanding Older Adults’ Medication Decision Making and Behavior: A Study on over-the-Counter (OTC) anticholinergic medications |
| 86 | Holsteyn, 2013 | The psychology of retirement: Coping with the transition from work |
| 87 | Howard & Howard, 2013 | Aging mind and brain: Is implicit learning spared in healthy aging? |
| 88 | Hurt et al., 2012 | Cognition, coping, and outcome in Parkinson's disease |
| 89 | Ingebrand, 2021 | People with dementia positioning themselves as learners |
| 90 | Jacobs-Lawson et al., 2016 | Age Differences in Information Use While Making Decisions |
| 91 | James, 2012 | The Impact of Health and Financial Literacy on Decision Making in Community-Based Older Adults |
| 92 | Jamieson , 2012 | Learning in Later Adulthood: Transitions and Engagement in Formal Study |
| 93 | Johs-Artisensi et al., 2016 | Development of a self-assessment tool to facilitate decision-making in choosing a long term care administration major |
| 94 | Kapp, 2014 | Older Persons and Compromised Decisional Capacity: The Role of Public Policy in Defining and Developing Core Professional Competencies |
| 95 | Kardos et al., 2016 | Age-Related Characteristics of Risky Decision-Making and Progressive Expectation Formation |
| 96 | Karlawish, 2008 | Measuring Decision-Making Capacity in Cognitively Impaired Individuals |
| 97 | Katsara & De Witte, 2019 | How to Use Socratic Questioning in Order to Promote Adults' Self-Directed Learning |
| 98 | Kavé et al., 2012 | Formal Education Level Versus Self-Rated Literacy as Predictors of Cognitive Aging |
| 99 | Khosravani et al., 2014 | Analyzing the Effects of Iranian EFL Textbooks on Developing Learners' Life Skills |
| 100 | Kim & Kim, 2015 | Elderly Korean learners' participation in English learning through lifelong education: Focusing on motivation and demotivation. |
| 101 | Kim & Song, 2018 | Medical Decision-Making for Adults Who Lack Decision-Making Capacity and a Surrogate: State of the Science |
| 102 | Kim et al., 2017 | Development and Validation of the Attitudes toward Education for Older Adults (AEOA) Scale |
| 103 | Kim, 2008 | Reviewing and Critiquing Computer Learning and Usage among Older Adults |
| 104 | Kimhi et al., 2012 | Elderly People Coping With the Aftermath of War: Resilience Versus Vulnerability |
| 105 | Klugar et al., 2016 | The Personal Active Aging Strategies of Older Adults in Europe: A Systematic Review of Qualitative evidence |
| 106 | Knoepke & Johnson, 2017 | Empowering Older Adults to Make Medical Decisions: Two Solutions & A Problem |
| 107 | Kogan et al., 2016 | Person-Centered Care for Older Adults with Chronic Conditions and Functional Impairment: A Systematic Literature Review |
| 108 | Kolva et al., 2018 | Assessing the Decision-Making Capacity of Terminally Ill Patients with Cancer |
| 109 | Kouzuki et al., 2020 | A program of exercise, brain training, and lecture to prevent cognitive decline |
| 110 | Krause, 2012 | Valuing the Life Experience of Old Adults and Change in Depressive Symptoms: Exploring an Overlooked Benefit of Involvement in Religion |
| 111 | Kyndt et al., 2014 | Development and validation of a questionnaire on informal workplace learning outcomes: A study among socio-educational care workers. |
| 112 | Lagacé et al., 2012 | The silent impact of ageist communication in long term care facilities: Elders' perspectives on quality of life and coping strategies |
| 113 | Lai & Karlawish, 2008 | Assessing the Capacity to Make Everyday Decisions: A Guide for Clinicians and an Agenda for Future Research |
| 114 | Lancaster & Carlson, 2015 | Meaning made, distress, and growth: An examination of the Integration of Stressful Life Experiences Scale. |
| 115 | Lekoko & Nthomang, 2018 | Lifelong learning for Africa's adults: The role of open educational resources and indigenous learning |
| 116 | Lelorain, 2012 | Posttraumatic growth in long term breast cancer survivors: Relation to coping, social support and cognitive processing. |
| 117 | Lev-Wiesel & Amir, 2003 | Postraumatic growth among holocause child survivors |
| 118 | Lhermite et al., 2019 | Decision-Making Capacity Among Elderly People: A Mapping of Health Professionals' and Laypeople's Views |
| 119 | Lichtenberg et al., 2018 | Conceptual and Empirical Approaches to Financial Decision- Making by Older Adults: Results from a financial decision-making rating scale |
| 120 | Lichtenberg et al., 2020 | A Short Form of the Lichtenberg Financial Decision Rating Scale |
| 121 | Lin, Li-Hui | The Social Outcomes of Older Adult Learning in Taiwan: Evaluation Framework and Indicators |
| 122 | Linehan et al., 2015 | Dialectical behavior therapy for high suicide risk in individuals with borderline personality disorder: a randomized clinical trial and component analysis |
| 123 | Lippa, 2012 | Coping With Behavior Problems in Patients with Dementia |
| 124 | Literat, 2014 | Measuring New Media Literacies: Towards the Development of a Comprehensive Assessment Tool |
| 125 | Löckenhoff et al., 2018 | Aging and Decision-Making: A Conceptual Framework for Future Research – A Mini-Review |
| 126 | Lühnen et al., 2019 | Informed decision-making with and for people with dementia: Developing and pilot testing an education program for legal representatives (PRODECIDE) |
| 127 | Lusardi, 2012 | Financial Literacy and Financial Decision-Making in Older Adults |
| 128 | Mao & Peck, 2013 | Assessment Strategies, Self-Regulated Learning Skills, and Perceptions of Assessment in Online Learning |
| 129 | Martin-Hammond et al., 2015 | Designing an Over-the-Counter Consumer Decision-Making Tool for Older Adults |
| 130 | Mason et al., 2013 | Promoting Intergenerational Interaction through Collaborative Learning: “Growing and Aging” Community Reading Program |
| 131 | McEwen et al., 2018 | Simultaneous Aerobic Exercise and Memory Training Program in Older Adults with Subjective Memory Impairments |
| 132 | McGovern et al., 2014 | Reward-related decision making in older adults: relationship to clinical presentation of depression |
| 133 | McLean et. al., 2013 | Posttraumatic growth and posttraumatic stress among military medical personnel. |
| 134 | McSwiggan et al., 2016 | Decision-making capacity evaluation in adult guardianship: a systematic review |
| 135 | Merriam & Kee, 2014 | Promoting community wellbeing: The case for lifelong learning for older adults. |
| 136 | Milana et al., 2017 | The Palgrave International Handbook on Adult and Lifelong Education and Learning |
| 137 | Mitoku et al., 2014 | The Decision-Making and Communication Capacities of Older Adults with Dementia: A Population-Based Study |
| 138 | Mountain et al., 2017 | A preventative lifestyle intervention for older adults (lifestyle matters): a randomised controlled trial |
| 139 | Moye & Marson, 2007 | Assessment of Decision-Making Capacity in Older Adults: An Emerging Area of Practice and Research |
| 140 | Mutter & Arnold, 2021 | Aging and associative binding in contingency learning |
| 141 | Narushima et al., 2013 | The Association Between Lifelong Learning and Psychological Well-Being Among Older Adults: Implications for Interdisciplinary Health Promotion in an Aging Society |
| 142 | Navarro et al., 2016 | Attitudes toward Science: Measurement and Psychometric Properties of the Test of Science-Related Attitudes for Its Use in Spanish-Speaking Classrooms |
| 143 | Nguyen, 2020 | Adaptation for Growth Via Learning New Skills as a Means to Long-Term Functional Independence in Older Adulthood: Insights From Emerging Adulthood |
| 144 | Obhi, 2021 | Values of lifelong learners and their pursuits of happiness and whole-person wellness |
| 145 | Ogletree & Blieszner, 2022 | Dark Times and Second Chances: Perceived Growth From Adversity |
| 146 | Ortiz et al., 2012 | Insightful learning of life's lessons with older adult guests in the classroom. |
| 147 | Pae, 2012 | A psychometric measurement model for adult English language learners: Pearson test of English academic. |
| 148 | Paillaud et al., 2017 | Preferences about information and decision-making among older patients with and without cancer |
| 149 | Papen, 2012 | Informal, incidental and ad hoc: The information-seeking and learning strategies of health care patients. |
| 150 | Pat‐El et al., 2013 | Validation of assessment for learning questionnaires for teachers and students. |
| 151 | Patrick et al., 2022 | Religious and Spiritual Growth Goals: A Forgotten Outcome |
| 152 | Peeters et al., 2014 | Adult learners’ informal learning experiences in formal education setting. |
| 153 | Peisah et al., 2013 | Decisional capacity: toward an inclusionary approach |
| 154 | Pennington et al., 2018 | Tools for testing decision-making capacity in dementia |
| 155 | Perach et al., 2021 | Emotion Regulation and Decision-Making in Persons with Dementia : A Scoping Review |
| 156 | PERCY, 2013 | Lifelong Learning in Later Life: A Handbook on Older Adult Learning |
| 157 | Petrillo et al., 2018 | Older Adult and Surrogate Perspectives on Serious, Difficult, and Important Medical Decisions |
| 158 | Pietrzak et al., 2012 | Mild Worry Symptoms Predict Decline in Learning and Memory in Healthy Older Adults: A 2-Year Prospective Cohort Study |
| 159 | Pizzi et al., 2014 | Cost-effectiveness of a community-integrated home-based depression intervention in older African Americans |
| 160 | Pooley et al., 2013 | Posttraumatic stress and posttraumatic growth and their relationship to coping and self-efficacy in Northwest Australian cyclone communities. |
| 161 | Powers et al, 2013 | Attitudes Toward Older Adults: Results from a Fitness-Based Intergenerational Learning Experience |
| 162 | Prat et al., 2013 | The impact of skills training on cognitive functioning in older people with serious mental illness |
| 163 | Price & Murray, 2012 | The Region of Proximal Learning Heuristic and Adult Age Differences in Self-Regulated Learning |
| 164 | Qualls & Smyer, 2007 | Changes in Decision Making Capacity in Older Adults |
| 165 | Quan-Haase et al., 2014 | Not All on the Same Page: E-Book Adoption and Technology Exploration by Seniors |
| 166 | Resnick, 2014 | Resilience in Older Adults |
| 167 | Rienzo, 2014 | Recognition and Validation of Non Formal and Informal Learning: Lifelong Learning and University in the Italian Context |
| 168 | Rodrigues et al., 2018 | Effectiveness of education at work in a health program: a strategy for vocational training and lifelong learning |
| 169 | Rolison et al., 2011 | Risky Decision Making in Younger and Older Adults : The Role of Learning Risky Decision Making |
| 170 | Romaioli & Contarello, 2021 | Resisting ageism through lifelong learning. Mature students' counter-narratives to the construction of aging as decline |
| 171 | Romo et al., 2016 | Decision Making among Older Adults at the End of Life: A Theoretical Perspective |
| 172 | Rosenfeld et al., 2016 | Age and life course location as interpretive resources for decisions regarding disclosure of HIV to parents and children: Findings from the HIV and later life study |
| 173 | Rosi et al., 2019 | Decision-Making Competence in Younger and Older Adults: Which Cognitive Abilities Contribute |
| 174 | Rowlands et al., 2017 | Health literacy and the social determinants of health: A qualitative model from adult learners. |
| 175 | Saade et al., 2018 | Attitudes towards continuous professional development: a study of pharmacists in Lebanon |
| 176 | Sablotny-Wackershauser, 2020 | Older Adults Show a Reduced Tendency to Engage in Context-Dependent Decision Biases |
| 177 | Samsi & Manthorpe, 2013 | Everyday decision-making in dementia: findings from a longitudinal interview study of people with dementia and family carers |
| 178 | Amaral et al., 2020 | Exploratory Study on Healthcare Decision-Making Capacity Assessment |
| 179 | Satake & Murray, 2014 | Teaching an Application of Bayes' Rule for Legal Decision-Making: Measuring the Strength of Evidence |
| 180 | Schepers et al., 2012 | Sense of Competence in Dementia Care Staff (SCIDS) scale: development, reliability, and validity |
| 181 | Schmidt, 2014 | Personal Growth as a Strong Element in the Motivation of Australian University Students to Learn German |
| 182 | Schoenmakers et al., 2012 | Coping with loneliness: What do older adults suggest? |
| 183 | Seaman et al., 2016 | Adult age differences in decision making across domains: Increased discounting of social and health-related rewards |
| 184 | Shakespeare-Finch et al., 2013 | A qualitative approach to assessing the validity of the posttraumatic growth inventory. |
| 185 | Sherwood et al., 2017 | Effects of Coping Skills Training on Quality of Life, Disease Biomarkers, and Clinical Outcomes in Patients With Heart Failure: A Randomized Clinical Trial |
| 186 | Sheykhi, 2012 | Ageing and the need of e-learning in Tehran, Iran: A sociological appraisal of elder development |
| 187 | Shibu et al., 2019 | Assessing Patients Decision-Making Capacity in the Hospital Setting : A Literature Review |
| 188 | Silva et al., 2012 | Pathways for psychological adjustment in breast cancer: A longitudinal study on coping strategies and posttraumatic growth. |
| 189 | Silverstein et al., 2018 | Problem-Solving Education to Prevent Depression Among Low-Income Mothers: A Path Mediation Analysis in a Randomized Clinical Trial |
| 190 | Simon & Gluck, 2013 | Adult Age Differences in Learning and Generalization of Feedback-Based Associations |
| 191 | Siqveland et al., 2012 | Posttraumatic growth in parents after a natural disaster. |
| 192 | Sloniger, 2013 | Functional fitness testing of older adults: An intergenerational service learning experience. |
| 193 | Stevenson et al., 2019 | Perception and Communication of Risk in Decision Making by Persons with Dementia |
| 194 | Stewart et al., 2020 | Loneliness Interacts with Cognition in Relation to Healthcare and Financial Decision Making |
| 195 | Stormoen et al., 2013 | Health and Disability Investigating Medical Decision-Making Capacity in Patients with Cognitive impairment using a protocol based on linguistic features |
| 196 | Suwa et al., 2018 | Developing a Questionnaire on User Needs and Decision-Making Regarding Home-Care Robots for Older People in Japan, Ireland and Finland |
| 197 | Tam, 2012 | Elder learning in Hong Kong: Policies, programmes, provisions, and issues. |
| 198 | Tam, 2012 | East-West Perspectives on Elder Learning |
| 199 | Tam, 2013 | A model of active ageing through elder learning: The Elder Academy Network in Hong Kong. |
| 200 | Tam, 2018 | Retirement and Learning: A Longitudinal Qualitative Approach |
| 201 | Tauber & Rhodes, 2012 | Multiple Bases for Young and Older Adults' Judgments of Learning in Multitrial Learning |
| 202 | Taubman-Ben-Ari et al., 2012 | Personal Growth and the Transition to Grandfatherhood |
| 203 | Thompson et al., 2020 | Exploring stress, coping, and decision-making considerations of Alzheimer's family caregivers |
| 204 | Tinetti et al., 2019 | Challenges and Strategies in Patients’ Health Priorities-Aligned Decision-Making for Older Adults with multiple chronic conditions |
| 205 | Tomás et al., 2012 | Resilience and coping as predictors of general well-being in the elderly: A structural equation modeling approach |
| 206 | Tomich & Helgeson, 2012 | Posttraumatic growth following cancer: Links to quality of life. |
| 207 | Toyama et al., 2020 | Psychosocial factors promoting personal growth throughout adulthood. |
| 208 | Trachsel et al., 2015 | Cognitive Fluctuations as a Challenge for the Assessment of Decision-Making Capacity in Patients With Dementia |
| 209 | Triplett et al., 2012 | Posttraumatic growth, meaning in life, and life satisfaction in response to trauma. |
| 210 | Usher & Stapleton, 2019 | Decision-Making Capacity Assessment: Occupational Therapy's Contribution within a Multidisciplinary Approach |
| 211 | Usher & Stapleton, 2020 | Approaches for Assessing Decision-Making Capacity in Older Adults: A Scoping Review Protocol |
| 212 | Usher & Stapleton, 2021 | Assessment of Older Adults’ Decision- ­making Capacity in Relation to Independent Living : A scoping review |
| 213 | Valer et al., 2015 | The significance of healthy aging for older persons who participated in health education groups |
| 214 | Van Patten & Bellone, 2021 | Appreciating cognitive and emotional growth in older adults |
| 215 | Walker-Williams et al., 2013 | Coping Behaviour, posttraumatic growth and psychological well-being in women with childhood sexual abuse. |
| 216 | Wallace et al., 2012 | Coping with health stresses and remission from late-life depression in primary care: a two-year prospective study |
| 217 | Wang & Christiansen, 2019 | An Investigation of Chinese Older Adults' Self-Directed English Learning Experience Using Mobile Apps |
| 218 | Ward, 2015 | Ageing and learning disability: Putting older people with learning disabilities on the map. |
| 219 | Wayde et al., 2017 | Decision-making quality of younger and older adults in familiar and unfamiliar domains |
| 220 | Weigold et al., 2013 | Examining tenets of personal growth initiative using the Personal Growth Initiative Scale–II. |
| 221 | Weir et al., 2018 | Decision-Making Preferences and Deprescribing: Perspectives of Older Adults and Companions About Their Medicines |
| 222 | Wolfson et al., 2014 | Older Adults and Technology- Based Instruction : Optimizing Learning Outcomes |
| 223 | Wong et al., 2014 | Empowerment of Senior Citizens via the Learning of Information and Communication Technology |
| 224 | Wood et al., 2020 | Decision-Making Capacity Evaluations : The Role of Neuropsychological Assessment from a Multidisciplinary perspective |
| 225 | Woods et al., 2021 | Perceived Social Support and Interpersonal Functioning as Predictors of Treatment Response Among Depressed Older Adults |
| 226 | Worthy, 2011 | With Age Comes Wisdom: Decision-Making in Younger and Older Adults |
| 227 | Wuif et al., 2012 | Altering Mindset Can Enhance Motor Learning in Older Adults |
| 228 | Xie et al., 2012 | Understanding and Changing Older Adults' Perceptions and Learning of Social Media |
| 229 | Yamashita et al., 2017 | Types of learning activities and life satisfaction among older adults in urban community-based lifelong learning programs. |
| 230 | Ye Li et al., 2013 | Complementary Cognitive Capabilities, Economic Decision Making, and Aging |
| 231 | Zarghami et al., 2018 | Using Multi-Criteria Decision-Making Method (MCDM) to Study Quality of Life Variables in the Design of Senior Residences in Iran |
| 232 | Zwart et al., 2019 | Procedural Learning across the Lifespan: A Systematic Review with Implications for Atypical development |
| 233 | Usher & Stapleton, 2022 | Assessing Older Adults’ Decision-Making Capacity for Independent Living: Practice Tensions and Complexities |
| 234 | Skelton et al., 2010 | Determining if an older adult can make and execute decisions to live safely at home: A capacity assessment and intervention model |
| 235 | Moye & Braun, 2010 | Decisional capacity assessment: Optimizing safety and autonomy for older adults |
| 236 | Shippee et al., 2015 | Supporting caregivers of older adults in making decisions: Current tools and future directions |
| 237 | Spike, 2009 | Assessment of decision-making capacity |
| 238 | van Bijsterveld et al., 2022 | Psychometric evaluation of the Decision Support Tool for Functional Independence in community-dwelling older people |
| 239 | Bush & Wood, 2021 | Determining decisional capacity across settings and clinical presentations: A systematic approach. |
| 240 | Moberg, 2005 | Decision-making capacity in the impaired older adult. |
| 241 | Amaral et al., 2021 | Decision-making capacity in healthcare: instruments review and reflections about its assessment in the elderly with cognitive impairment and dementia |
| 242 | Weigold et al., 2021 | College as a growth opportunity: Assessing personal growth initiative and self-determination theory |
| 243 | Mäkikangas et al., 2016 | Long-term development of employee well-being: a latent transition approach |
| 244 | Geise, 2008 | Personal growth and personality development: Well-being and ego development |
| 245 | Diener et al, 2009 | New Well-being Measures: Short Scales to Assess Flourishing and Positive and Negative Feelings |
| 246 | Yilmaz & Kaygin, 2018 | The Relation between Lifelong Learning Tendency and Achievement Motivation. |
| 247 | Meerah et al., 2011 | Measuring life-long learning in the Malaysian Institute of Higher Learning context |
| 248 | Solmazand & Aydim, 2016 | Evaluation of lifelong learning tendencies of pre-service teachers |
| 249 | Wielkiewicz & Meuwissen, 2014 | A lifelong learning scale for research and evaluation of teaching and curricular effectiveness |
| 250 | Chen & Liu, 2019 | The different style of lifelong learning in China and the USA based on influencing motivations and factors |
| 251 | Solmaz, 2017 | Relationship between Lifelong Learning Levels and Information Literacy Skills in Teacher Candidates. |
| 252 | Feiz & Hooman, 2013 | Assessing the Motivated Strategies for Learning Questionnaire (MSLQ) in Iranian students: Construct validity and reliability |

**Appendix 3: Potential measures / Questionnaire**

*Lifelong Learning Scale from Kirby et al., 2010. Development of a scale to measure lifelong learning.*

What are your opinions on learning?

| 1 | 2 | 3 | 4 | 5 |
| --- | --- | --- | --- | --- |
| Strongly disagree |  |  |  | Strongly agree |

| **Item** | **Opinions** |
| --- | --- |
| 1 | I prefer to have others plan my learning |
| 2 | I prefer problems for which there is only one solution |
| 3 | I can deal with the unexpected and solve problems as they arise |
| 4 | I feel uncomfortable under conditions of uncertainty |
| 5 | I am able to impose meaning upon what others see as disorder |
| 6 | I seldom think about my own learning and how to improve it |
| 7 | I feel I am a self-directed learner |
| 8 | I feel others are in a better position than I am to evaluate my success as a student |
| 9 | I love learning for its own sake |
| 10 | I try to relate academic learning to practical issues |
| 11 | I often find it difficult to locate information when I need it |
| 12 | When I approach new material, I try to relate it to what I already know |
| 13 | It is my responsibility to make sense of what I learn at school |
| 14 | When I learn something new I try to focus on the details rather than on the ‘big picture’ |

*Personal Growth and Development Scale from Anderson et al., 2019. Measuring Personal Growth and Development in Context: Evidence of Validity in Educational and Work Settings*

Instructions: Using the scale provided, tick on the most applicable circle for each statement to agreement

| 1 | 2 | 3 | 4 | 5 | 6 | 7 |
| --- | --- | --- | --- | --- | --- | --- |
| Not at all |  |  |  |  |  | Very much so |

My university experience so far has helped me…

| **Item** | **Questions** |
| --- | --- |
|  | **Autonomy** |
| 1 | Gain the strength to stand up for what I believe** |
| 2 | Feel confident in my decisions* |
| 3 | Appreciate the value of setting my own direction life |
|  | **Environmental mastery** |
| 4 | Learn how to manage my life more effectively |
| 5 | Gain confidence to deal with unforeseen difficulties* |
| 6 | Take advantage of opportunities in my surroundings** |
|  | **Positive relations** |
| 7 | Learn how to develop meaningful relationships with others** |
| 8 | Appreciate others’ perspectives on issues |
| 9 | Learn to work more effectively with others* |
|  | **Self-acceptance** |
| 10 | Feel more comfortable with who I am |
| 11 | Appreciate my strengths* |
| 12 | Feel good about the experiences that have shaped me** |
|  | **Purpose in life** |
| 13 | Discover what gives meaning to my life** |
| 14 | Identify important goals I want to achieve |
| 15 | Get close to understanding what I want out of life* |

*Note*. Items were represented without headings (e.g. Autonomy) and in a randomized order.

Items indicated with an ** recommended for a short, 5-item version of the PGDS while items indicated with an * are recommended, in addition to items included in the 5-item version, for the 10-item version.

*Decision making instrument from Karlawish & Lai, 2009. The Short Portable Assessment of Capacity for Everyday Decision-Making (ACED).*

**To administer the ACED you need to identify a functional problem the person is having, and at least one option to solve that problem. You will then adapt the interview questions according to the functional problem the person is experiencing and the options to take care of that problem. For each question, decide whether the person’s answer is adequate, marginal, or inadequate using the following scoring criteria:**

| **Understanding Scoring Criteria** | **Appreciation Scoring Criteria** | **Reasoning Scoring Criteria** |
| --- | --- | --- |
| **0 (inadequate performance):** Person gives a clearly inaccurate response with serious distortion.  **1 (marginal performance):**  Person shows some recollection of the item content but gives and incomplete and vague response.  **2 (adequate performance):**  Person recalls the content of the item and offers a fairly clear version of it. | **0:** Person offers reasons that are delusional or a serious distortion of reality or cannot answer the question.  **1:** Person may or may not believe the option will benefit/adversely affect his or her situation but the reason is vague and may represent distorted versions of reality.  **2:** Person acknowledges at least some potential benefit/adverse affect from the option and offers reasons that have some reasonable basis. | **Comparative:**  **0:** Person provides no comparative statements or an illogical one.  **1:** Person provides comparison statement without specific consequence.  **2:** Person provides clear comparison statement with specific consequences.  **Consequential:**  **0:** Person provides no everyday consequences or an illogical answer.  **1:** Person provides a general statement without details.  **2:** Person provides a clear and vivid statement of everyday consequences. |

The scoring criteria are to help guide your judgment whether the person has sufficient decisional abilities to make their own choice.

**What is the functional problem here? Fill in here:** _______________________________________

_________________________________________________________________________________

**What are some options to solve the problem? Fill in at least one here:**

|  |  |  |
| --- | --- | --- |
|  |  |  |

**Does the person understand the problem?**

Describe the functional problem the person is experiencing. Ask the person to say this back in his/her own words. Describe the consequences of the problem. Ask the person to say this back in his/her own words.

**Expressing a Choice**

Final choice to manage the functional problem. *“Now that we’ve had a chance to talk about [functional problem] what would you like to do?”*

**Consequential Reasoning**

What would happen if the person had to choose another option? *“How would [insert option to deal with functional problem] affect your everyday life?”*

**Comparative Reasoning**

How is the person’s choice better than another option (such as not getting help)? *“What makes your choice better than [state another option]?”*

**Does the person appreciate the benefits and downsides of the options?**

Does the person think that one of the options to manage the problem will benefit him/her? *“Now just consider this choice [restate an option]. Do you think [insert option to deal with functional problem] could benefit you?”*

Does the person think that an option might make things worse for him/her? “Now just consider this choice [restate an option]. *Do you think [insert option to deal with functional problem] might make things worse for you?”*

**Does the person understand the disadvantages of the option?**

Describe the disadvantages to the options. Ask the person to say this back in his/her words.

**Does the person understand the advantages of the option?**

Describe the advantages to the options. Ask the person to say this back in his/her words.

**Does the person understand the options to manage the problem?**

Describe the options to manage the functional problem. Ask the person to say this back in his/her own words.

**Does the person appreciate the problem?**

Does the person believe that he/she has the problem you described? *“Do you have any problems with the [state the functional problem]?”*
